# The First Quarter Century of the Dense Alignment Surface Transmembrane Prediction Method

**DOI:** 10.3390/ijms241814016

**Published:** 2023-09-13

**Authors:** Miklós Cserző, Birgit Eisenhaber, Frank Eisenhaber, Csaba Magyar, István Simon

**Affiliations:** 1 Institute of Enzymology, Research Centre for Natural Sciences, 1117 Budapest, Hungary; cserzo.miklos@med.semmelweis-univ.hu (M.C.); magyar.csaba@ttk.hu (C.M.); 2 Department of Physiology, Faculty of Medicine, Semmelweis University, 1094 Budapest, Hungary; 3 Bioinformatics Institute, Agency for Science, Technology and Research (A*STAR), Singapore 138671, Singapore; birgit@eisenhaber.org (B.E.); frank@eisenhaber.org (F.E.); 4 Genome Institute of Singapore, Agency for Science, Technology and Research (A*STAR), Singapore 138671, Singapore; 5 LASA—Lausitz Advanced Scientific Applications gGmbH, 02943 Weißwasser, Germany; 6 School of Biological Sciences, Nanyang Technological University (NTU), Singapore 637551, Singapore

**Keywords:** transmembrane proteins, transmembrane prediction, multiple sequence alignment, dot-plots

## Abstract

The dense alignment surface (DAS) transmembrane (TM) prediction method was first published more than 25 years ago. DAS was the one of the earliest tools to discriminate TM proteins from globular ones and to predict the sequence positions of TM helices in proteins with high accuracy from their amino acid sequence alone. The algorithmic improvements that followed in 2002 (DAS-TMfilter) made it one of the best performing tools among those relying on local sequence information for TM prediction. Since then, many more experimental data about membrane proteins (including thousands of 3D structures of membrane proteins) have accumulated but there has been no significant improvement concerning performance in the area of TM helix prediction tools. Here, we report a new implementation of the DAS-TMfilter prediction web server. We reevaluated the performance of the method using a five-times-larger, updated test dataset. We found that the method performs at essentially the same accuracy as the original even without any change to the parametrization of the program despite the much larger dataset. Thus, the approach captures the physico-chemistry of TM helices well, essentially solving this scientific problem.

## 1. Introduction

The majority of transmembrane (TM) proteins with known three-dimensional structures have helical TM segments [1] (available online: http://pdbtm.enzim.hu/ accessed on 30 June 2023), which are built up from approximately 15–30 residues with the dominancy of hydrophobic amino acids. Transmembrane proteins are abundant; about 20–30% of the proteins encoded in the human genome are TM proteins [2]. TM proteins have numerous functions in living cells: they can participate in regulation and intercellular communication by forming receptors on cell surfaces, or can form transport channels through plasma membranes, to name a few [3]. Most of the target proteins of currently approved drug molecules are in fact TM proteins [4]; thus, they are an especially interesting class of proteins from a medical aspect.

TM proteins are difficult to produce by recombinant protein-expression systems. Likewise, their experimental structure determination is a complicated task, too [5]. This fact contributed largely to the fast development of in silico theoretical methods dealing with TM proteins. There have been several TM prediction methods developed by research groups worldwide. One of the earliest methods was TOPPRED [6], which predicted a relatively high number of false positive (FP) hits. Several other successful prediction methods were developed in the next decade, like MEMSAT [7], PHD [8], and TMHMM [9]. There were also additional TM prediction methods created in the Institute of Enzymology, like the recent CCTOP method [10] based on the consensus of different TM prediction methods, which is currently among the best performing TM prediction methods.

The development of TM topology-prediction methods is unbroken. It is even accelerating, with the huge leap forward in the use of deep-learning-based methods in the life sciences [11]. The number of experimentally solved TM protein structures has increased significantly in the last 20 years [1], which provided a good basis for deep-learning-based TM prediction methods, like TMBED [12], and DeepTMHMM [13]. All of these methods are able to predict the topology of TM proteins based on their amino acid sequence with very high accuracy.

**Figure 1:**
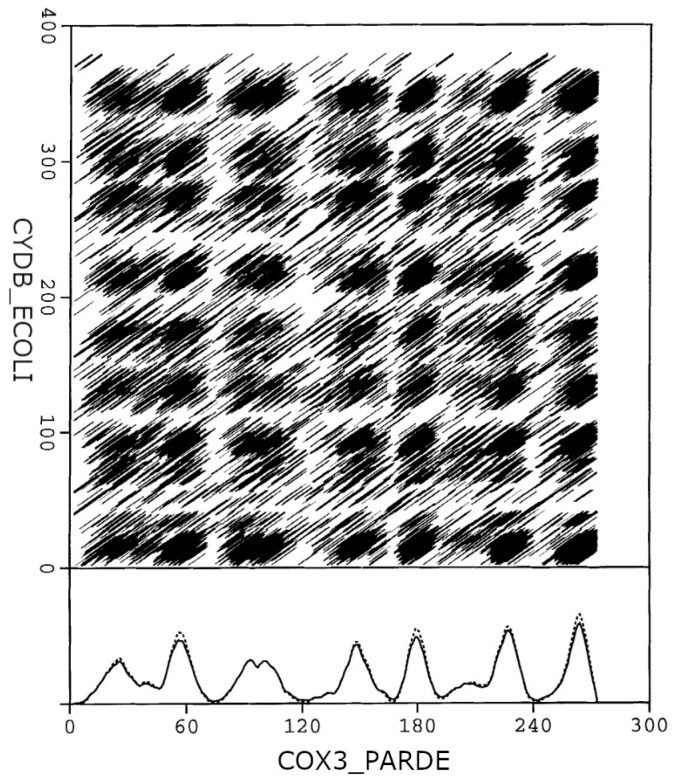
DAS plot of two proteins (COX3_PARDE vs CYDB_ECOLI). The cross-weighted cumulative score profile (dotted line) and the global DAS profile (continuous line) calculated as the average of the cumulative score profiles, obtained from comparison with the other proteins in the test set, is shown for COX3_PARDE.

The dense alignment surface (DAS) transmembrane analysis algorithm [14] is a simple method published originally in 1997, which is able to identify helical TM segments in proteins. The algorithm is based on pairwise sequence alignments using a special scoring matrix. This substitution table scores the similarity of the amino acids from the viewpoint of hydrophobicity. As transmembrane helices are predominantly hydrophobic, any TM segment is similar to any other TM segment in this respect. Moreover, the similarity remains even after shifting the two segments relative to each other. Consequently, intersections of TM segments appear as black squares on the graphical representation of the alignment surface. This chessboard-like pattern correlates with the location of the reported TM segments. Projecting the alignment surface to the axes results in high precision hydrophobicity curves for the two sequences used in the process. This process is demonstrated in Figure 1, with the example of COX3_PARDE–CYDB_ECOLI pairwise sequence alignment. 

The algorithm uses a library of experimentally well-documented sets of TM proteins for the reference. The query sequence is compared to these in a pairwise fashion (for details see the original publication [15]). The method does not need any information or assumption about the investigated TM protein; the only used information is encoded in a small TM-protein sequence library.

In 2002, the DAS-TMfilter method was introduced as a successor of the original DAS, which was able to filter out false positive (FP) TM-protein predictions more effectively [15]. The method was tested on a dataset of 128 experimentally validated TM proteins, and was found to perform at a high recall precision of 96%. In the last 25 years, the DAS and the DAS-TMfilter methods have been used by thousands and performed more than one hundred thousand predictions, while the two publications have received more than 1200 citations. The original public web pages of the DAS and DAS-TMfilter methods became unavailable recently, with the exception of the server at https://mendel.imp.ac.at/DAS/ (accessed on 30 June 2023) which is still up and running. The growth of the TM dataset in the last 20 years has given us the opportunity to reevaluate the performance of the prediction method and modernize the web-server side of the code at the same time.

## 2. Results

**Figure 2:**
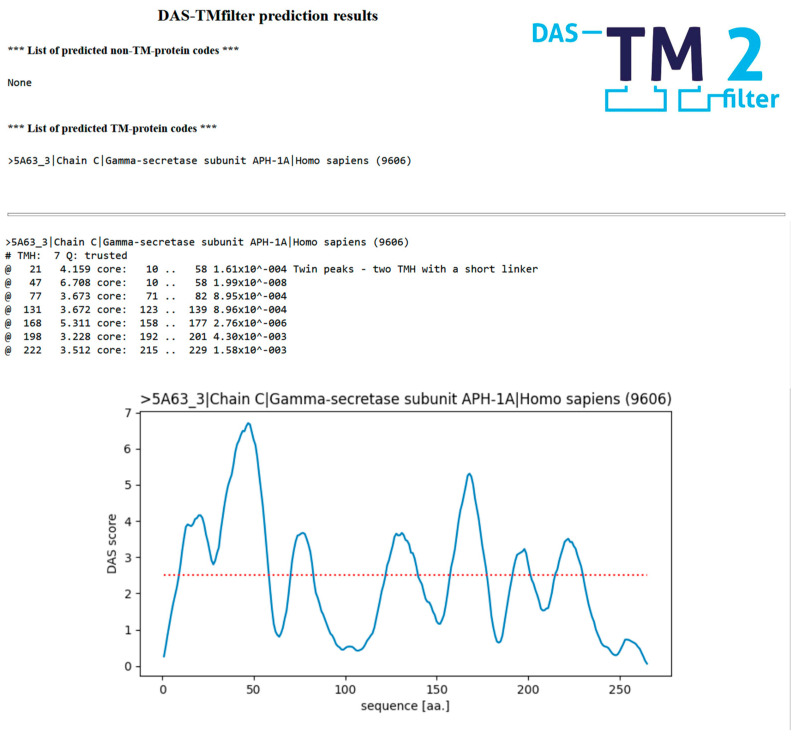
Example output obtained for the human gamma-secretase protein prediction, using the protein-sequence information available in the 5A63 entry of the PDB database. The red dotted line shows the cutoff value of 2.5 for the DAS score, which is used to identify TM helical regions.

The original, unmodified code of the DAS-TMfilter method was implemented as a web server by wrapping the original Linux executable binary in Python source using the “Bottle python web framework” as user interface. The new server is accessible under the public https://das.enzim.ttk.hu (accessed on 30 June 2023) web address. We benchmarked the new web server using the recommended library size (8) on modern commodity Intel CPUs, and we found that approximately 10 predictions could be performed in one second. There is a choice for short-text-only output and for long output, including the figures of the DAS score profiles. An example of the server long output for the amino acid sequence of the human gamma-secretase protein with the 5A63 [16] Protein Data Bank (PDB) [17] identifier can be seen in Figure 2. The webpage offer a number of choices. There is a choice of free and fixed options for figure scaling, where the fixed option uses a cutoff value of 5 for the DAS profile scores, to make the comparison of DAS profiles of different proteins easier by using the same fixed scale. There is also a choice between unconditional and trusted evaluation options. In unconditional predictions, a quality value is always calculated. Using the trusted option, the method decides automatically if calculation of the quality value is necessary. The quality value is important for queries with just a single predicted TM segment, influencing the decision about whether the query protein is TM or not. There is also a choice of the size of the TM library (8, 16, 24, 32) used for the calculations. We suggest the use of the smallest (8) sized library; the choice of larger library sizes is kept for backwards-compatibility reasons. 

This new implementation of the DAS-TMfilter algorithm was tested on the dataset used by Bernhofer and Rost, which was downloaded from their GitHub repository (https://github.com/BernhoferM/TMbed/tree/main/data (accessed on 30 June 2023)) as referred to by the supplement therein [12]. Briefly, these sequence collections are supported by experimental evidence, checked and cross referenced in relevant databases, filtered in several steps, and contain homology reduced sequences only: 593 α-helical TM proteins, 65 β-barrel TM proteins, and 5859 water-soluble non-TM proteins. The sequences are accompanied by a crude topology description (annotation) based on a six-stage model: helical (“H/h”), beta (“B/b”), signal (“S”), intracellular (“1”), extracellular (“2”), and unknown/unresolved (“U”).

Since the DAS method can be used to predict TM helical segments, the 593 helical subset of this dataset was used (Table S1). The authors performed a detailed comparison of several TM prediction methods (see Per-Segment Performance data in the publication of Bernhofer and Rost [12]). We evaluated our method using the recall and the precision performance evaluation metrics using almost the same methodology, and have found that our 25-year-old method performs on-par with the latest deep-learning-based methods In Table 1 we can see the per-segment performance of our method on the original [15], and the new dataset.

Taking a closer look beyond the overall performance we identified 2882 true positive (TP), 190 false positive, and 136 false negative (FN) hits, resulting in a recall value of 0.955, and a precision value of 0.938, respectively. In the case of the false negative hits, in most cases (105 of 136) only a single TM segment is missing from the prediction. Two TM segments are missing in 19 predictions, three segments are missing in 9 predictions, 4 segments are missing in 2 predictions, and there is a single prediction which misses 6 TM segments.

An even closer look reveals 32 examples, with at least one false positive hit and one false negative hit in the same sequence. Visual inspection of this small set of sequences reveals a few problems with the decision-making mechanism. For example, in the case of SC6A9_HUMAN (UNIPROT [18] accession P48067), two consecutive TM helices are annotated with a short linker (“twin peaks”) between residues 499 and 546 (structures 6ZBV and 6ZPL [19]). Our method also predicts two TM segments, but due to the slight shift, the second segment, as in the 3D structure annotation, overlaps with the first, predicted TM. Thus, the first predicted segment is linked to the two annotated segments and the second one is incorrectly classified as FP, when this false FP is in fact a TP. Generally, the handling of “twin peaks” is problematic due to the high number of possibilities in the overlap of annotated and predicted TMs. The algorithm gives a warning for consecutive TMs with a short linker (the “twin peak warning;” see Figure 2).

In case of O66528_AQUAE (O66528, 6FV6 [20]), the annotated peak between residues 19 and 38 is matched by the prediction, but the shoulder of the predicted peak is counted as a second segment separated by a single residue without annotation overlap. This results again in a false FP, which is actually a TP. These problems of short linkers cannot be easily solved with the proper decision-making rules, since these rules would be rather complicated and based on a low number of examples. The algorithm gives warnings instead, and lets the user make the final decision.

For the KDPA_ECOLI (P03959, 5MRW [21] and 6HRA [22]) sequence, a TM annotated from 357 to 368 in the structure is close to the predicted TM between 376 and 398. Similar small shifts of predicted TMs relative to TMs annotated in membrane protein 3D structures might be within the error of the experimental technics [15]. If so, that would mean the elimination of an FP and an FN at the same time. Thus, after some manual fine-tuning of the prediction results, the overall performance could be even higher than the already high reported values for the fully automated run.

Additionally to the per-segment performance we also evaluated the per-protein TM identification performance of our method using a globular non-TM (Table S2), and signal peptide (Table S3) datasets, used by Bernhofer and Rost [12]. Results can be seen in Table 2. 

In the case of the previously described TM dataset, our method identifies 95.1% of the entries correctly as TM. The non-TM globular protein dataset of Bernhofer and Rost [12] was divided in two parts, based on the signal peptide (SP) content of the proteins. On the globular dataset without SPs, our method identifies 91.9% of the entries correctly as non-TM. On the SP containing dataset, our method identifies about half of the entries as TM proteins, while the other half appears as non-TM. The presence of signal peptides confuses our method, but it could also be possible that our method identifies membrane-embedding signal peptides correctly and there is an additional type of signal peptide. These “targeting” peptides could have a different mode of action; they might be, for example, ligands of signal receptors. However, membrane-embedding signal peptides are functionally not real TM segments; biophysically, they are correctly identified as TM helices, and are just cleaved off during protein translocation. Fortunately, this weakness of the method can be easily compensated for by the application of a signal peptide prediction pre-filtering step.

The original DAS-TMfilter used reference libraries of different sizes. The smallest library, with 8 reference sequences, already provides good results. The optimization on the smaller test set suggested that performance could be improved with libraries containing 16, 24, or 32 sequences at the expense of linearly increasing CPU time. This possibility has been checked using the recent database but did not work; we could not see an improvement justifying the slower running of the code. The full version of the prediction test is bulky and accessible at https://das.enzim.ttk.hu/performance.html (accessed on 30 June 2023).

Another possible source of FP predictions has been demonstrated in the example of prion protein [23]. The algorithm detects a strong TM segment in sequence, while the prion protein is not membrane-related in its native form. However, under pathological conditions the structure turns inside out, exposing the hydrophobic core. This change initiates plaque formation and its related medical conditions. In this case, the prion protein can make hydrophobic interactions via the predicted segment. Strong FP predictions could be considered from this respect, too.

There is one difference between our analysis and that of Bernhofer and Rost [12]: how we identify hits. During the development of the original DAS method, it was apparent that the experimental data are not accurate at the per-residue level. This is a straightforward consequence of the extreme difficulties of experimental investigations on TM proteins. On the one hand, the experimental conditions for the membrane proteins used in structure determination cause strain and potential distortions of the native structures. This will affect also the protein regions at the membrane–cytoplasm boundary. On the other hand, the structural dynamics of membrane-embedded parts of the structure are not well understood and the identity of the residues at the membrane–cytoplasm boundary is most likely not constant over time. Therefore, the endpoints cited in the experimental data should not considered the real entry points of the protein TM segments into the membrane. As such, the data are not suitable as a target for any prediction-optimization procedure. Consequently, over-strict criteria are not practical for correct hits.

The other aspect is the length of the TM segment, which is generally expected to stretch 15 to 30 residues. The TM segments form bundles in the membrane, and the outside of the bundle contacts a lipid and is therefore hydrophobic. However, on the inside of the bundle, helix-to-helix interactions dominate and these inter-bundle parts are not necessarily hydrophobic. The importance of this effect grows with the number of helices in the bundle. This also holds for proteins with a low number of TM segments, if the functional structure involves oligomeric arrangement of the monomers. The final functional assembly contains a large number of TM segments in this case. As our algorithm essentially evaluates the hydrophobicity of localized amino acid patterns in the sequence, even very short predicted segments can be real hits. Thus, the employment of over-strict criteria for predicted TM segment length appears unpractical.

Because of the considerations above, we did not aim at perfect prediction of the TM helices on the residue level, and did not exclude excessively short hits. We did not use the same two-level criteria for identifying correct TM segment predictions as described by Bernhofer and Rost [12]. 

We classified a TM segment as a correct prediction when the structural annotation and the prediction have an overlap of at least three residues. Since on average 91% of the residues in the predicted segments were annotated as TM helices, this definition does not introduce a bias in the performance measurement of our prediction, and our data are directly comparable with the data of Bernhofer and Rost [12] in their Table 1 and Table 2.

One more difference needs to be pointed out. Signal peptides are not differentiated from helical TM segments by our method, and they were not considered false positive hits during the performance evaluation. Functionally, signal peptides are not real TM segments, but biophysically they are correctly identified as transmembrane helices. These helices are cleaved off from the protein during the maturation process, which can be taken as a post-translational-modification step. We believe that this is not a serious shortcoming in our method. Since there are several in silico methods for the identification of signal peptides [24], we did not intend to develop an additional signal peptide identification method. Our method only gives a warning message of “possible signal peptide” when a peak on the DAS score plot is observed within the first 25 residues of a protein (see Figure 1). If the user would not like to identify signal peptides as transmembrane helices, the removal of the signal peptide is recommended before performing the actual DAS prediction, for example, with the SignalP 6.0 method [24].

The performance of the method was also tested on the β-barrel set of Bernhofer and Rost, but these segments were not detected by the DAS-TMfilter algorithm. This was no surprise, because beta TM segments have different structural organization properties, and from its development onward our method was intended to detect α-helical TM segments. Since helical TM proteins account for almost 94% of all known TM 3D structures [1], this fact does not reduce the usability of our method significantly. 

## 3. Discussion

There has been a great increase in the number of experimentally solved TM protein structures in the last 25 years. According to the PDBTM database [1], there are currently more than 8700 TM protein structures present in the PDB, while the database starting size back in 2003 was merely 337. The performance of the DAS TM prediction method was reevaluated using a five-times-larger, non-redundant test. Using the recall and precision metrics, we obtained a per-segment value of 0.955, and 0.938, respectively. This means that the unmodified DAS method performs very well, staying among the top ranked prediction methods. This proves that the basic idea behind the DAS method is still valid 25 years after its development. The underlying biophysical theory has managed to withstand the test of time; it describes well the structural organization of transmembrane proteins. The detailed analysis of the false positive hits showed, furthermore, that the real life performance of the DAS method could be even higher, if the automated analysis is complemented by human visual inspection. Although there is only a little room for improvement, we are currently investigating if the utilization of a protein-size-dependent cutoff value could be used on the hydrophobicity curves in an automated way to further improve the prediction performance, by reducing the “twin peaks” problem. 

Since the beginning of the field of TM prediction, there has always been competition between methods that are based on the physico-chemical properties of localized amino acid patterns in protein sequences (DAS-TMfilter is one example) and machine learning tools. Notably, the latter require orders of magnitude more adjustable parameters than the former approaches and, thus, are significantly more dependent on the size and quality of the learning set. The surprising results of the reevaluation of the DAS-TMfilter presented in this work demonstrate that, despite the five-times-larger dataset used in testing, the prediction accuracy remains at the same high level as in the original publication. Thus, the parametrization from more than two decades ago captured some of the physico-chemical essence of TMs. Therefore, we can assume that the optimum of prediction tools relying on local sequence features has been reached and no further revolutionary development can be expected in this specific field. Further improvement will require the involvement of features distant in the sequence as well as of environmental factors (such as the vicinity of membranes as in the case of 1COL PDB entry [25]). The newly coded web engine for the DAS-TMfilter will provide long-term sustainability to the program and WWW server as well as compatibility with current software standards.

## 4. Materials and Methods

The evaluation datasets were downloaded from the GitHub page of TMBED [12] (https://github.com/BernhoferM/TMbed/tree/main/data/datasets (accessed on 30 June 2023)). The “alpha.fasta” file contained 593 α-helical TM protein sequences. The “signal.fasta” file contained 632 non-TM protein sequences, which contain a signal peptide, and 5161 non-TM protein sequences without signal peptides. One entry was deleted from the list of signal-peptide-containing proteins, because our program sporadically produced errors using it. The deleted entry was the unusual fibroin heavy chain precursor (P05790) sequence, which contains several hundred GA, GS, and GY repeats.

The performance of our method was evaluated using recall and precision performance metrics on the 593-element helical TM protein dataset on a per-segment level using the standard definitions based on the numbers of true positive (TP) hits (number of correctly identified TM helices), false negative (FN) hits (number of incorrectly not identified TM helices), and false positive (FP) hits (number of incorrectly identified TM helices).

A predicted TM segment was classified as a true positive hit when the predicted and the structure-derived topology available in the test dataset had an overlap of at least three residues. Because of this definition, even in the case of true positive hits, the predicted and the annotated TM segments can have slightly different lengths. Signal peptides were not considered during the identification of TM segments; they were not considered false positive hits.

## Figures and Tables

**Table 1:** Transmembrane protein segment identification performance.

	Original Dataset	New Dataset
Number of proteins	128	593
Number of annotated TM segments	618	3018
Number of predicted TM segments	615	3072
True positives	588	2882
False positives	27	190
False negatives	30	136
Recall	0.951	0.955
Precision	0.956	0.938

**Table 2:** Transmembrane protein identification performance.

	TM Dataset	Globular Dataset (without Signals)	Signal Peptides
Total number of entries	593	5161	631
	100%	100%	100%
Number of entries identified as TM	564	419	303
	95.1%	8.1%	48%
Number of entries identified as non-TM	29	4742	328
	4.9%	91.9%	52%

## Data Availability

The results of the predictions performed at the test dataset can be found at das.enzim.ttk.hu/performance.html (accessed on 30 June 2023) webpage.

## Supplementary Materials

The supporting information can be downloaded at: https://www.mdpi.com/1422-0067/24/18/14016/s1.
