# Correction: Cserző et al. The First Quarter Century of the Dense Alignment Surface Transmembrane Prediction Method. *Int. J. Mol. Sci.* **2023**, *24*, 14016

**DOI:** 10.3390/ijms25063422

**Published:** 2024-03-18

**Authors:** Miklós Cserző, Birgit Eisenhaber, Frank Eisenhaber, Csaba Magyar, István Simon

**Affiliations:** 1 Institute of Enzymology, Research Centre for Natural Sciences, 1117 Budapest, Hungary; cserzo.miklos@med.semmelweis-univ.hu (M.C.); magyar.csaba@ttk.hu (C.M.); 2 Department of Physiology, Faculty of Medicine, Semmelweis University, 1094 Budapest, Hungary; 3 Bioinformatics Institute, Agency for Science, Technology and Research (A*STAR), Singapore 138671, Singapore; birgit@eisenhaber.org (B.E.); frank@eisenhaber.org (F.E.); 4 Genome Institute of Singapore, Agency for Science, Technology and Research (A*STAR), Singapore 138671, Singapore; 5 LASA—Lausitz Advanced Scientific Applications gGmbH, 02943 Weißwasser, Germany; 6 School of Biological Sciences, Nanyang Technological University (NTU), Singapore 637551, Singapore

There was an error in the original publication [1]. The eighth sentence of the second paragraph of the Introduction (“Despite the higher accuracy of CCTOP, it cannot be used for binary classification, i.e., to establish whether a protein is TM or not.”) is not valid for the CCTOP method. It has to be deleted together with the last sentence of the same paragraph (“The DAS-TMfilter is one of the few methods which can do this accurately.”). The corrected paragraph is as follows:

TM proteins are difficult to produce by recombinant protein-expression systems. Likewise, their experimental structure determination is a complicated task, too [5]. This fact contributed largely to the fast development of in silico theoretical methods dealing with TM proteins. There have been several TM prediction methods developed by research groups worldwide. One of the earliest methods was TOPPRED [6], which predicted a relatively high number of false positive (FP) hits. Several other successful prediction methods were developed in the next decade, like MEMSAT [7], PHD [8], and TMHMM [9]. There were also additional TM prediction methods created in the Institute of Enzymology, like the recent CCTOP method [10] based on the consensus of different TM prediction methods, which is currently among the best performing TM prediction methods.

The authors state that the scientific conclusions are unaffected. This correction was approved by the Academic Editor. The original publication has also been updated.
